# Dobutamine stress testing for the evaluation of atrial and diastolic ventricular function in Fontan patients

**DOI:** 10.1136/openhrt-2020-001487

**Published:** 2021-03-12

**Authors:** Jelle P G van der Ven, Sjoerd S M Bossers, Eva van den Bosch, Niels Dam, Irene M Kuipers, Gabrielle G van Iperen, Lucia J M Kroft, Livia Kapusta, Arend D J ten Harkel, Willem A Helbing

**Affiliations:** 1 Pediatric Cardiology, Erasmus MC Sophia Children Hospital, Rotterdam, Zuid-Holland, The Netherlands; 2 Netherlands Heart Institute, Utrecht, Utrecht, The Netherlands; 3 Department of Pediatrics, Division of Cardiology, Amsterdam UMC Locatie AMC, Amsterdam, North Holland, The Netherlands; 4 Department of Pediatrics, Division of Cardiology, UMC Utrecht, Utrecht, Utrecht, The Netherlands; 5 Department of Radiology, LUMC, Leiden, Zuid-Holland, The Netherlands; 6 Department of Pediatrics, Division of Cardiology, Radboudumc, Nijmegen, Gelderland, The Netherlands; 7 Pediatric Cardiology Unit, Tel Aviv Sourasky Medical Center, Tel Aviv, Israel; 8 Department of Paediatric Cardiology, LUMC, Leiden, Zuid-Holland, The Netherlands; 9 Paediatric Cardiology and Radiology, Erasmus MC Sophia Children Hospital, Rotterdam, Zuid-Holland, The Netherlands

**Keywords:** magnetic resonance imaging, fontan procedure, heart defects, congenital

## Abstract

**Objective:**

To assess the atrial and ventricular diastolic function response to dobutamine stress in Fontan patients, and to relate these measurements to exercise capacity and events during the follow-up.

**Methods:**

We performed a secondary analysis of a cross-sectional multicentre study of Fontan patients with intra-atrial lateral tunnel (ILT) or extracardiac conduit (ECC) modification. Subjects underwent cardiac MRI during rest and low-dose dobutamine stress, and cardiopulmonary exercise testing. Atrial and diastolic ventricular function parameters were derived from volume-time curves.

Medical records were abstracted for a composite end-point of death, listing for transplant, arrhythmia and reintervention. Spearman’s r correlation tests and Cox proportional hazards models were used to assess the relation between the dobutamine response for atrial and diastolic ventricular function and outcomes, including exercise capacity.

**Results:**

We included 57 patients (26 ECC; 31 ILT) aged 12.8 (IQR (10.3–15.5)) years. During dobutamine stress atrial cyclic volume change increased (3.0 (0.4–5.9) mL/m^2^, p<0.001), as did early (1.9 (−1.6 to 3.6) mL/m^2^, p=0.001) and late emptying volume (2.2 (0.2–4.4) mL/m^2^, p<0.001).

Ventricular early filling decreased (−1.6 (−5.7 to 0.7) mL/m^2^, p=0.046) and ventricular late filling increased (1.0 (−0.4 to 3.4) mL/m^2^, p<0.001) while stroke volume remained similar.

Only for patients with the ECC modification, atrial early emptying volume increase correlated with peak oxygen uptake (ρ=0.66, p=0.002). No other parameter related to exercise capacity.

During a median 7.1-year follow-up, 22 patients reached the composite endpoint. No parameter predicted events during the follow-up.

**Conclusions:**

Dobutamine stress augmented atrial reservoir and pump function for Fontan patients. Atrial early emptying reserve related to exercise capacity in ECC patients. No other atrial or diastolic ventricular function parameter related to outcomes.

**Key questions**

**What is already known about this subject?**

- Fontan patients are generally limited by impaired single ventricular inflow, but the role of the atria in ventricular filling has received little attention.

**What does this study add?**

- Atrial reservoir and pump function increase with dobutamine stress. Higher atrial early emptying volume reserve relates to higher exercise capacity in extracardiac conduit patients.

**How might this impact on clinical practice?**

- Stress testing of the pulmonary venous atrium might demonstrate occult dysfunction in Fontan patients.

## Introduction

The Fontan strategy is the preferred treatment for univentricular heart defects.^1^ In the Fontan circulation, blood is ejected by the single ventricle into the aorta and systemic vasculature. Through the Fontan connection blood flows passively over the pulmonary vasculature, without the presence of a subpulmonary pump. Although this palliation results in (near) normal aortic oxygen saturation and eliminates single ventricular volume overload, the unique physiology of a Fontan circulation can result in a myriad of complications in the long term, including arrhythmia, thromboembolism, protein losing enteropathy and plastic bronchitis.^1^ In the common concept of the Fontan circulation the ‘bottle-necks’ in this circulation include (1) potentially suboptimal flow in the baffle connecting the inferior caval vein to the pulmonary arteries, (2) limited pulmonary venous return to the single ventricle because of passive pulmonary flow with abnormal pulsatility, (3) inflow impairment into the single ventricle related to diastolic dysfunction and (4) systolic single ventricular dysfunction.^2 3^ Many patients have normal systolic function yet impaired circulatory function. Ventricular inflow impairment relates to several factors including, among others, ventricular diastolic properties and atrial function.^4 5^ Remarkably the contribution of the atria in the Fontan circulation has been poorly studied.^1^

Previously we demonstrated that atrial and ventricular diastolic function at rest—measured by cardiac MRI (CMR)—are not related to outcomes in Fontan patients.^5^ Patients with a Fontan circulation have an abnormal diastolic function response to dobutamine stress, compared with age-related controls.^6–8^ This results in a decrease of single ventricle end diastolic volume (EDV) during the dobutamine stress.^9^ Imaging during dobutamine stress can be used to unmask occult dysfunction^10^ and has been linked to clinical outcomes.^11^ In this study, we aimed to assess atrial and ventricular diastolic function during rest and dobutamine stress CMR in Fontan patients and relate the dobutamine stress response to exercise capacity and events during the follow-up. We hypothesise the atrial and ventricular response to dobutamine stress allows for a better characterisation of single ventricular inflow impairment than measurements obtained during a resting state and relates to clinical outcome.

## Methods

### Study design

We performed a secondary analysis of a Dutch multicentre cross-sectional study.^9^ Inclusion criteria for this previous study were: (1) Fontan patients aged ≥8 year; (2) having undergone a staged total cavopulmonary connection (TCPC) with either intra-atrial lateral tunnel (ILT) or extracardiac conduit (ECC); (3) TCPC completion before 7 years of age; (4) at least 3 years of follow-up since completion TCPC. Exclusion criteria were severe intellectual impairment or contraindications for CMR. All subjects or their legal guardians provided written informed consent. Patients or the public were not involved in the design or execution of the study. For the current study, all subjects who successfully underwent dobutamine stress CMR with complete imaging of the atria and cardiopulmonary exercise testing were included for analysis.

### CMR study

All patients underwent steady state free precession (SSFP) CMR on a 1.5T whole-body scanner. Images were obtained in either the short axis or axial orientation including complete imaging of the atria. An extended CMR protocol has been published previously.^10^ After CMR acquisition during resting conditions, the CMR study was repeated with the same imaging protocol during low-dose (7.5 µg/kg/min) intravenous dobutamine infusion.^10^ Vital parameters were monitored continually during dobutamine infusion. Imaging protocol was resumed once a new steady state in vital parameters was achieved. Dobutamine infusion was reduced to 5 µg/kg/min in case of adverse events (ie, an increase in heart rate, systolic or diastolic blood pressure of ≥50% compared with baseline or a decrease in heart rate, systolic or diastolic blood pressure of ≥20%, or if the subject experienced discomfort). If adverse events persisted, dobutamine infusion was halted.

### CMR postprocessing

Segmentation was performed on a Windows workstation using Medis Suite 3.2 (Medis Medical Imaging Systems, Leiden, the Netherlands). Epicardial contours of the single ventricle were manually segmented in all phases and slices. All ventricular structures that eject in the aorta were segmented as a functional single ventricle. Endocardial segmentation of the ventricle was performed automatically by a threshold-based method (MassK, Medis Medical Imaging Systems, Leiden, the Netherlands) and manually adjusted where needed. All atrial structures that drain pulmonary venous return and unload in the single ventricle were manually segmented, considering them as a functional mono-atrium. Examples of segmentation are provided in online supplemental files. Atrioventricular connections are classified according to the sequential segmental analysis categorisation by Anderson and Shirali.^12^ Functional parameters were derived from atrial and ventricular volume-time curves, as previously described.^5^ The reserve of a functional parameter is defined as ‘dobutamine stress value minus resting value’. Functional parameters are summarised in table 1. Volumes are indexed for body surface area, derived using the Haycock *et al* formula.^13^

10.1136/openhrt-2020-001487.supp1 Supplementary video

10.1136/openhrt-2020-001487.supp2 Supplementary video

**Table 1:** Ventricular and atrial functional parameters

Parameter	Definition
*Ventricle*	
Early filling volume (mL/m^2^)	Ventricular filling volume during the first 1/3 of diastole
Late filling volume (mL/m^2^)	Ventricular filling volume during atrial contraction
Deceleration time (ms)	Time from early peak filling rate to zero-velocity baseline, based on the linear segment of the downward slope
Early peak filling rate (mL/s)	Peak filling rate during early diastole
Late peak filling rate (mL/s)	Peak filling rate during late diastole
E/A ratio	Early peak filling rate/late peak filling rate
*Atria*	
Maximal volume (mL/m^2^)	
Minimal volume (mL/m^2^)	
Cyclic volume change (mL/m^2^)	Maximal volume—minimal volume
Early emptying volume (mL/m^2^)	Atrial emptying volume during early diastole, considered to reflect atrial reservoir function.
Late emptying volume (mL/m^2^)	Atrial emptying volume during late diastole, considered to reflect atrial contractile—or booster pump—function.
Conduit volume (mL/m^2^)	Ventricular filling can occur without atrial volume change, that is, when venous return equals atrioventricular flow. This conduit volume is calculated using the following formula: Ventricular stroke volume (atrial early emptying volume+atrial late emptying volume).

All volumes are indexed for body surface area.

### Outcomes

Exercise capacity and events during the follow-up were assessed. All subjects underwent cardiopulmonary exercise testing on the same day as CMR examination, as previously described.^14^ Peak oxygen uptake (averaged over the final 30 s of testing) was assessed for patients with maximal exercise capacity (defined as respiratory exchange ratio (RER) ≥1.05). Ventilatory efficiency (VE/CO2) slope during submaximal exercise was assessed for all patients.

Patient records were abstracted for events during the follow-up. The day of study visit was considered day 0 for follow-up. Events were assessed as a composite endpoint, consisting of: death; listing for heart transplant; arrhythmia (defined as any arrhythmia requiring medication or intervention); and reintervention (both surgical and catheter-based reinterventions).

### Statistical analysis

Continuous data are presented as median (IQR). Continuous data were compared using the non-parametric Wilcoxon test. Paired tests were used for the comparison of rest and stress measurements. Categorical data are presented as count (percentage) and compared using the χ^2^ test or Fisher’s exact test for tests including counts of zero. Correlations were assessed by the non-parametric Spearman’s ρ. Events during follow-up were assessed by Cox proportional hazards models. A p<0.05 was considered statistically significant. All statistical tests with regard to outcome were adjusted for multiple testing using the Benjamini-Hochberg procedure. All analyses were performed using R V.3.6.1 (R Core Team, 2019, Vienna, Austria).

## Results

### Patient characteristics

We identified 57 unique patients with complete examinations. Patient characteristics are shown in table 2. Twenty-six patients with an ECC Fontan modification and 31 patients with an ILT Fontan modification were included. Median age was 12.8 years (range 8.4–26.3).

**Table 2:** Patients characteristics

	Study population	ECC	ILT	P value
N	57	26	31	
Sex (male)	37 (65%)	15 (41%)	22 (59%)	0.296
Age at study (years)	12.8 (10.3–15.5)	11.9 (10.7–14.6)	13.6 (10.1–16.6)	0.228
Age at Fontan (years)	2.9 (2.5–3.7)	3.1 (2.5–3.8)	2.9 (2.5–3.6)	0.594
BSA (m^2^)	1.34 (1.15–1.59)	1.37 (1.19–1.51)	1.28 (1.13–1.74)	0.867
Dominant ventricle				0.174
Left	39 (68%)	20 (77%)	19 (61%)	
Right	17 (30%)	5 (19%)	12 (39%)	
Undetermined	1 (2%)	1 (4%)	0 (0%)	
Cardiac diagnosis				
TA	13 (23%)	9 (35%)	4 (13%)	0.052
DILV	10 (18%)	3 (12%)	7 (23%)	0.275
DORV	10 (18%)	2 (8%)	8 (26%)	0.073
PA +IVS	7 (12%)	4 (15%)	3 (10%)	0.513
HLHS	7 (12%)	3 (12%)	4 (13%)	0.876
Other	10 (18%)	5 (19%)	5 (16%)	0.759
Heterotaxy syndrome	7 (12%)	3 (12%)	4 (13%)	0.876
AV connection type				0.089
Concordant	20 (35%)	9 (35%)	11 (35%)	
Discordant	0 (0%)	0 (0%)	0 (0%)	
Two atria to one ventricle	16 (28%)	4 (15%)	12 (39%)	
One atrium to two ventricles	19 (33%)	11 (42%)	8 (26%)	
Ambiguus	2 (4%)	2 (8%)	0 (0%)	
AV valve regurgitation				0.882
None to trivial	40 (70%)	18 (69%)	22 (71%)	
Moderate to severe	17 (30%)	8 (31%)	9 (29%)	
VE/VCO_2_ slope (%)	108.7 (101.3–118.7)	106.0 (101.5–112.3)	112.2 (101.5–148.6)	**0.042**
RER≥1.05 (n)	37 (65%)	20 (77%)	17 (55%)	0.082
Peak oxygen uptake (% predicted)	81.5 (66.2–93.7)	82.7 (67.6–88.4)	78.1 (65.4–96.0)	0.918

Characteristics are shown for the study population and for both surgical modifications. Exercise capacity is shown as percentage of predicted value. P values reflect the difference between surgical modifications.

AV, atrioventricular; DILV, double inlet left ventricle; DORV, double outlet right ventricle; ECC, extracardiac conduit; HLHS, hypoplastic left heart syndrome; ILT, intra-atrial lateral tunnel; MA, mitral atresia; PA+IVS, pulmonary atresia with intact ventricular septum; RER, respiratory exchange ratio; TA, tricuspid atresia; VE/VCO_2_, ventilatory efficiency.

Thirty-nine patients had a dominant left ventricle (LV), 17 had a dominant right ventricle (RV) and 1 patient had a single ventricle of undetermined morphology. Patients with an LV and RV had similar age (13.2 (10.5–15.6) for LV vs 12.8 (10.3–15.5) for RV, p=0.737) and body surface area (BSA) (p=0.215).

Moderate-to-severe AV regurgitation was present in 17 (30%) of patients. The presence of AV regurgitation did not differ between surgical modifications (p=0.882).

Exercise capacity in the study population was relatively well preserved. Maximal exercise effort (RER ≥1.05) was achieved in 37 patients. For these patients peak oxygen uptake was 82% (66 – 94) of the predicted value for age.

### Atrial response to dobutamine stress

Atrial cyclic volume change increased from 9.7 (7.5–12.7) to 13.5 (10.1–17.0) mL/m^2^ during dobutamine stress (p<0.001). Atrial early emptying volume increased by 1.9 (−1.6 to 3.6] mL/m^2^, p=0.001. Atrial late emptying volume increase by 2.2 (0.2–4.4) mL/m^2^ (p<0.001). Conduit volume did not change during dobutamine stress (p=0.092). For ECC patients atrial maximal volume decreased during dobutamine stress (difference −3.3 (−11 to 1.5) mL/m^2^), whereas ILT patients increased maximal volume by 2.7 (−0.6 to 6.9) mL/m^2^ (p=0.001 for difference between ILT and ECC). Similarly, atrial conduit volume increased for ECC patients during dobutamine stress and decreased for ILT (2.7 (−2.7 to 6.5) vs −4.7 (−9.2 to −0.5) mL/m^2^ change, p=0.003).

No parameter of atrial function reserve correlated with heart rate increase. Atrial response to dobutamine stress did not differ between patients with a dominant LV versus RV. Resting atrial minimal volume was higher in patients with moderate-to-severe AV regurgitation compared with those without. No differences in atrial function (reserve) was seen between patients with and without significant AV regurgitation or across groups of AV connections.

### Ventricular response to dobutamine stress

Parameters of atrial and ventricular parameters during rest and dobutamine stress are shown in table 3. During the dobutamine infusion, ventricular EDV and end-stroke volume (ESV) both decreased. Stroke index remained similar and cardiac output increased as a result of higher heart rates with dobutamine infusion (as previously reported).^9^ During the dobutamine stress, ventricular early filling volume slightly decreased and atrial contraction related (late) ventricular filling volume increased. E/A ratio decreased from 1.5 (1.2–1.8) to 1.2 (0.9–1.6). Deceleration time did not change with dobutamine stress (neither did deceleration time/RR duration). This was true for either surgical modification (p=0.125 for difference between ILT and ECC).

**Table 3:** Ventricular and atrial parameters during rest and dobutamine stress

Parameters	Rest	Dobutamine	Reserve	P value
*Ventricle*				
Heart rate (/min)	71.4 (62.1–87.1)	97.1 (77.4–112.1)	20.0 (6.0–30.7)	**<0.001**
End-diastolic volume (mL/m^2^)	96.8 (80.0–130.0)	88.4 (72.6–111.8)	−7.5(−15.5–−1.9)	**<0.001**
End-systolic volume (mL/m^2^)	49.3 (37.8–67.1)	38.1 (30.6–47.7)	−8.9(−19.5–−4.6)	**<0.001**
Stroke volume (mL/m^2^)	49.1 (38.6–62.2)	53.5 (39.2–61.8)	2.1 (−2.1–5.8)	0.060
Ejection fraction (%)	49.3 (42.5–55.6)	57.6 (48.5–62.9)	6.8 (3.6–14.2)	**<0.001**
Deceleration time (ms)	177(134–240)	176(131–241)	−7(−77–73)	0.664
Early filling volume (mL/m^2^)	13.6 (8.8–18.9)	10.1 (6.6–16.1)	−1.6(−5.7–0.7)	**0.046**
Atrial filling volume (mL/m^2^)	9.8 (7.6–13.0)	10.9 (9.4–13.8)	1.0 (−0.4–3.4)	**<0.001**
Early peak filling rate (mL/s)	194(142–254)	221(170–272)	30 (−1–62)	**<0.001**
Late peak filling rate (mL/s)	134(99–172)	192(136–251)	39(17–106)	**<0.001**
E/A ratio	1.5 (1.2–1.8)	1.2 (0.9–1.6)	−0.2(−0.5–0.1)	**0.001**
*Atria*				
Maximal volume (mL/m^2^)	34.9 (28–48.3)	37.1 (28.1–46.1)	1.4 (−5.4–4.7)	0.949
Minimal volume (mL/m^2^)	25.6 (18.8–32.8)	22.0 (18.0–30.3)	−2.8(−8.5–2.4)	**0.018**
Cyclic volume change (mL/m^2^)	9.7 (7.5–12.7)	13.5 (10.1–17.0)	3.0 (0.4–5.9)	**<0.001**
Early emptying volume (mL/m^2^)	6.9 (5.3–9.1)	8.4 (7.2–10.9)	1.9 (−1.6–3.6)	**0.001**
Late emptying volume (mL/m^2^)	6.5 (4.8–8.8)	8.9 (7.1–12.4)	2.2 (0.2–4.4)	**<0.001**
Conduit volume (mL/m^2^)	21 (15.7–27.1)	17.9 (12–24.7)	−2.5(−7.6–3.6)	0.092

All volumes are indexed for body surface area.

Lower ventricular early filling volume reserve and higher early peak filling rate reserve were related to higher heart rate increase during dobutamine stress (p<0.015). No other parameters were related to heart rate increase. During dobutamine stress patients with a dominant RV increased their stroke volume more than patients with a dominant LV (4.5 (1.9–8.6) vs −0.2 (−3.9 to 2.6) mL/m^2^, p=0.001). Conversely, patients with a dominant LV increased their heart rate more than patients with a dominant RV (24.1 (11.7–32.2) vs 16.5 [(1.0–23.7) bpm, p=0.027). Patients with a dominant LV had a larger decrease of early filling volume during dobutamine stress, compared with those with a dominant RV (−3.7 (−7.2 to 0.2) vs −1 (−2.6 to 1.5) mL/m^2^, p=0.043)

Patients with moderate-to-severe AV regurgitation had higher ventricular stroke volume reserve compared with those without. No other parameter of ventricular function differed between patients with and without significant AV regurgitation. Diastolic ventricular function (reserve) did not differ across groups of AV connections.

### Predictors of exercise capacity

Predictors of peak oxygen uptake and VE/VCO2 slope are shown in table 4. Higher atrial early emptying volume reserve correlated with higher peak oxygen uptake (ρ=0.37, p=0.026), but this correlation was not considered statistically significant after adjusting for multiple testing. Stratified by Fontan modifications, only for ECC patients higher atrial early emptying volume reserve correlated with higher peak oxygen uptake (r=0.66, p=0.002). This association remained statistically significant after adjusting for multiple testing. No other functional parameter predicted peak oxygen uptake. No functional parameter predicted VE slope. Peak heart rate during exercise testing weakly correlated with heart rate during (low-dose) dobutamine stress (r=0.30, p=0.023), but also with heart rate during rest (r=0.32, p=0.014).

**Table 4:** Correlations between functional reserve parameters and exercise capacity

	Peak oxygen uptake		VE/VCO_2_	
	r	P value	r	P value
*Ventricle*				
End-diastolic volume reserve	0.34	0.039	−0.20	0.143
End-systolic volume reserve	0.10	0.560	−0.02	0.879
Stroke volume reserve	0.25	0.139	−0.21	0.115
Ejection fraction (%)	0.01	0.959	−0.20	0.137
Early filling volume reserve	0.06	0.706	−0.16	0.243
Atrial filling volume reserve	0.11	0.501	−0.18	0.172
Deceleration time (ms)	−0.01	0.943	0.19	0.176
E/A ratio	0.06	0.743	−0.04	0.775
*Atria*				
Maximal volume reserve	0.05	0.756	0.14	0.315
Minimal volume reserve	−0.08	0.626	0.11	0.407
Cyclic volume change reserve	0.25	0.134	0.01	0.969
Early emptying volume reserve	0.37	0.026	0.05	0.719
Late emptying volume reserve	0.10	0.551	−0.09	0.517
Conduit volume reserve	−0.08	0.648	−0.18	0.184

Reserve is defined as the difference between measurements during dobutamine stress and resting values. All volumes are presented as mL/m^2^, indexed for body surface area. P values are shown unadjusted for multiple testing. No P values were considered statistically significant after adjusting for multiple testing.

### Predictors of events during follow-up

During a 7.1 (6.0–8.2) years follow-up 22 patients (39%) experienced a clinical event. Two patients died during the follow-up. Five patients underwent reoperation and an additional seven patients underwent interventional procedures. Thirteen patients experienced arrhythmia (12 supraventricular tachycardia; 1 third-degree AV block). One-year event-free survival estimate was 91% (84%–99%). Five years event-free survival estimate was 75% (95% CI 64% to 87%). Predictors of events during the follow-up are shown in table 5. No parameter of atrial or ventricular diastolic function predicted events during the follow-up.

**Table 5:** Predictors of events during follow-up

	HR	P value
*Ventricle*		
End-diastolic volume reserve	0.98 (0.93 to 1.03)	0.437
End-systolic volume reserve	1.02 (0.95 to 1.08)	0.647
Stroke volume reserve	0.95 (0.89 to 1.01)	0.113
Ejection fraction reserve (%)	0.95 (0.89 to 1.02)	0.148
Early filling volume	1.00 (0.91 to 1.11)	0.939
Atrial filling volume	0.88 (0.77 to 1.01)	0.070
Deceleration time reserve (ms)	1.00 (1.00 to 1.00)	0.862
E/A ratio reserve (unitless)	1.18 (0.62 to 2.27)	0.612
*Atria*		
Maximal volume reserve	1.00 (0.95 to 1.04)	0.888
Minimal volume reserve	1.00 (0.95 to 1.06)	0.908
Cyclic volume change reserve	0.98 (0.89 to 1.07)	0.608
Early emptying volume reserve	1.03 (0.90 to 1.17)	0.700
Late emptying volume reserve	1.04 (0.92 to 1.18)	0.508
Conduit volume reserve	0.94 (0.88 to 1.00)	0.043

Cox proportional hazards models were used for survival analysis. Reserve is defined as the difference between measurements during dobutamine stress and resting values. All volumes are presented as mL/m^2^, indexed for body surface area. P values are shown unadjusted for multiple testing. No P values were considered statistically significant after adjusting for multiple testing.

## Discussion

We assessed atrial function and diastolic ventricular function at rest and during dobutamine stress in a cohort of young staged TCPC patients. We noted that atrial early and late emptying increased during the dobutamine stress CMR, rather than conduit volume. For ECC patients atrial early emptying volume reserve related to peak oxygen uptake. This has not been demonstrated at rest.^5^ We could not demonstrate a relationship between the type of morphology of the atria and atrioventricular connection with atrial, diastolic ventricular function or clinical outcome. No other parameters of ventricular or atrial function related to either exercise capacity or clinical events during the follow-up.

The atria are important to provide adequate drainage of pulmonary venous blood in the, often preload impaired, single ventricle in the Fontan circulation. As different complex congenital heart diseases can necessitate a Fontan palliation, the morphology and connections of the atria is highly variable in this patient population. Atrial isomerism, double inlet or absent AV connections and one or two AV valves, including hypoplastic AV annuli are all part of the morphological spectrum of univentricular hearts. This might affect the haemodynamic relationship between the atria and both the pulmonary venous drainage and inflow into the single ventricle. In this study and with current methodology, we could not demonstrate an effect of dominant ventricular morphology or type of AV connection.

Different surgical modifications of TCPC manipulate the atria in different ways. Atrial scarring as a result of surgery might disturb the compliance and pump function of the atrium. The ECC bypasses the atria completely, whereas the ILT modification creates a baffle in the right atrium using either prosthetic or tissue material.^1^ This excludes part of the atrial volume to create a connection between the inferior caval vein and the pulmonary artery. It has been suggested that the ILT technique might facilitate synergistic movements between the pulmonary venous atrium and Fontan baffle.^15^ At the same time, this technique might also limit size related function of the pulmonary venous atrium. Previously, we demonstrated ILT patients have preserved atrial volumes of the pulmonary venous draining atrium, despite having part of the atrial volume used to create the intra-atrial tunnel.^5^ In the current study, atrial maximal volume increased during dobutamine stress for ILT patients, while it decreased for ECC patients. This might be related to the effects of dobutamine on the part of the (contractile) atrial wall forming the intra-atrial tunnel.

Little is known about the normal atrial response to dobutamine stress. During exercise atrial, early and late emptying increase.^16 17^ Trained athletes have a higher increase of atrial late emptying following exercise compared with sedentary controls.^17^

Power loss in the Fontan circuit has been studied extensively, but the pulmonary venous atrium has received little attention in this respect.^15 18 19^ Future studies that would assess flow dynamics and power loss in the pulmonary venous atrium would be of great interest. Energetically favourable flow patterns through the atria are probably necessary for a good early ventricular filling phase in the context of diminished pulmonary venous return. A better understanding of atrial (dys)function could be used to guide treatment strategies for patients with functionally univentricular congenital heart disease.

In long-standing diastolic dysfunction in structurally normal hearts, atrial pressure increases to maintain early ventricular filling.^20^ This is reflected in atrial size and function.^21^ Patients with heart failure with preserved ejection fraction have diminished atrial early and late emptying compared with healthy controls.^21^ Fontan patients, in contrast, generally have increased atrial pump function compared with health controls.^22 23^ We found dobutamine stress CMR reveals signs of (limited) impairment of diastolic single ventricular function, confirming earlier observations.^9 24^ Diastolic dysfunction of the single ventricle following Fontan palliation has previously been demonstrated using invasive measurements.^6 7 25 26^ At rest the isovolumetric relaxation constant (τ) is prolonged in Fontan patients compared with controls.^7^ During dobutamine stress, EDV decreases and end diastolic pressure remains similar.^6 7 25 26^ Isovolumetric relaxation shortens (as in healthy controls). In our present study, early peak filling rate and deceleration time, which are non-invasive estimates of isovolumetric relaxation, showed only a minor improvement during dobutamine stress. The ventricular stiffness constant β fails to decrease during dobutamine stress.^6–8^ In young Fontan patients, signs of impaired relaxation have been observed at rest.^7^ Dobutamine stress has been used to reveal diastolic dysfunction.^7 25 26^ Our present study provides non-invasive measures of diastolic function for Fontan patients during rest and dobutamine stress.

In previous research we demonstrated that the single ventricular reserve during low-dose dobutamine stress, a measure of systolic function, relates to clinical events during long-term follow-up.^11^ Despite the negative results of our current study evaluating occult atrial and ventricular diastolic function, dobutamine stress for the evaluation of occult systolic dysfunction may be a useful tool in the clinical evaluation of (asymptomatic) Fontan patients in adolescence and early adulthood. Stressors other than dobutamine, such as physiological exercise or lusotropic agents, could be considered for the evaluation of occult diastolic dysfunction in future research.

### Limitations

Several limitations of our present study should be considered. The lower temporal resolution of SSFP CMR, compared with echocardiography, can lead to underestimation or overestimation of parameters of diastolic function.^27^ We assessed the response to dobutamine stress using the same CMR protocol for both measurements to reduce the effect of temporal undersampling on stress reserve parameters.

Invasive measurements of atrial and ventricular pressure were not assessed due to the retrospective and non-invasive nature of our study. Non-invasive estimates of ventricular filling pressures, such as echocardiographic E/e’ were well within the normal range in a previous study including similar patients.^9^

Dobutamine stress is considered to mimic exercise physiology. However, exercise-induced changes in respiration and activity of the lower extremities muscles could affect ventricular filling in the Fontan circulation. As respiration has a considerable effects on ventricular filling, imaging was obtained during free breathing. The contribution of respiration to ventricular filling is not exacerbated during the exercise.^28^ During supine exercise CMR EDV and ESV fail to increase, similar to our results, implying a limited role of these factors in augmenting preload.^29 30^

We assessed a large number of both atrial and ventricular parameters for multiple clinical outcomes. To minimise the probability of type 1 errors (‘false positive findings’) corrections for multiple testing were applied. Despite the relatively large sample size, the penalty for multiple testing with a large number of parameters hampered statistical power. Future research replicating our results could confirm associations which were considered statistically significant before applying this penalty.

## Conclusions

We assessed the function of the pulmonary venous atrium and diastolic function of the single ventricle during resting conditions and dobutamine stress for Fontan patients of contemporary surgical modifications. Dobutamine stress increases atrial early and late emptying, rather than conduit volume. Atrial early emptying volume reserve relates to exercise capacity for patients with an ECC Fontan modification. No other parameters of atrial or ventricular diastolic function predicted outcomes. Atrial stress testing might demonstrate occult dysfunction and increase insight in ventricular inflow impairment seen in these patients.
